# Targeting SLC1A5 and SLC3A2/SLC7A5 as a Potential Strategy to Strengthen Anti-Tumor Immunity in the Tumor Microenvironment

**DOI:** 10.3389/fimmu.2021.624324

**Published:** 2021-04-19

**Authors:** Marianna Nachef, Alaa Kassim Ali, Saeedah Musaed Almutairi, Seung-Hwan Lee

**Affiliations:** ^1^ Department of Biochemistry, Microbiology and Immunology, Faculty of Medicine, University of Ottawa, Ottawa, ON, Canada; ^2^ School of Medicine, University College Dublin, Belfield, Dublin, Ireland; ^3^ Botany and Microbiology Department, College of Sciences, King Saud University, Riyadh, Saudi Arabia; ^4^ The University of Ottawa Centre for Infection, Immunity, and Inflammation, Ottawa, ON, Canada

**Keywords:** natural killer cells, anti-tumor immunity, nutrient transporters, immunometabolism, tumor microenvironment, SLC1A5, SLC7A5, SLC3A2

## Abstract

Cancer cells are metabolically vigorous and are superior in the uptake of nutrients and in the release of the tumor microenvironment (TME)-specific metabolites. They create an acidic, hypoxic, and nutrient-depleted TME that makes it difficult for the cytotoxic immune cells to adapt to the metabolically hostile environment. Since a robust metabolism in immune cells is required for optimal anti-tumor effector functions, the challenges caused by the TME result in severe defects in the invasion and destruction of the established tumors. There have been many recent developments in NK and T cell-mediated immunotherapy, such as engineering them to express chimeric antigen receptors (CARs) to enhance tumor-recognition and infiltration. However, to defeat the tumor and overcome the limitations of the TME, it is essential to fortify these novel therapies by improving the metabolism of the immune cells. One potential strategy to enhance the metabolic fitness of immune cells is to upregulate the expression of nutrient transporters, specifically glucose and amino acid transporters. In particular, the amino acid transporters SLC1A5 and SLC7A5 as well as the ancillary subunit SLC3A2, which are required for efficient uptake of glutamine and leucine respectively, could strengthen the metabolic capabilities and effector functions of tumor-directed CAR-NK and T cells. In addition to enabling the influx and efflux of essential amino acids through the plasma membrane and within subcellular compartments such as the lysosome and the mitochondria, accumulating evidence has demonstrated that the amino acid transporters participate in sensing amino acid levels and thereby activate mTORC1, a master metabolic regulator that promotes cell metabolism, and induce the expression of c-Myc, a transcription factor essential for cell growth and proliferation. In this review, we discuss the regulatory pathways of these amino acid transporters and how we can take advantage of these processes to strengthen immunotherapy against cancer.

## Introduction

Tumorigenesis, or the formation of a tumor, arises from multiple genetic alterations, mostly of oncogenes and tumor-suppressive genes, in normal cells that cause them to transform into neoplastic cells. These genetic alterations lead to major changes in the metabolism of cancer cells. The tumor microenvironment (TME), consisting of cellular and non-cellular elements, provides support for the growth and survival of tumor cells, and contribute to the resistance against the invasion of non-supportive cells, including cytotoxic lymphocytes. Tumor cells become metabolically hyperactive and increase their uptake of the nutrients within the TME. In addition, they release specific immunosuppressive cytokines such as transforming growth factor-β (TGF-β), which inhibits the metabolism of immune cells (1, 2). Altogether, the crosstalk between tumors and the TME elements, as well as the reduction in the availability of nutrients and oxygen, and the release of immunosuppressive metabolites such as lactate, generate a hostile environment (3–5). The combined immunosuppressive conditions of the TME are detrimental to tumor-infiltrating lymphocytes (TILs), which need to compete with cancer cells for nutrients to support their anti-tumor functions (1).

There are many recent advances in the development of novel immunotherapies to treat cancer, with a particular focus in enhancing the ability of immune cells to infiltrate and recognize the tumor. Promising results were obtained through immune checkpoint blockades, enhancing antibody-dependent cellular cytotoxicity (ADCC), as well as the adoptive transfer of genetically engineered NK or T cells (6, 7). Currently, numerous research teams aim to engineer NK or T cells to express chimeric antigen receptors (CARs), which combines the specificity of antibodies with the signaling capability of activating receptors, to redirect anti-tumor specificity and enhance homing to the tumor site (6, 7). Despite the generation of novel immunotherapies, immune cells remain at a disadvantage in the nutrient deficient and hostile TME, especially in solid tumors. To maintain their effector function as activated immune cells, avoid exhaustion, and continue protein synthesis for expansion in the TME, the expression of nutrient transporters on immune cells, specifically glucose and amino acid transporters, need to be increased and sustained to support the enhanced anabolic pathways. In humans, there are 458 members of the Solute Carrier (SLC) membrane-bound transporter in 65 families (8). They have important physiological roles since they allow for the transport of nutrients, drugs, and other small molecules. Among numerous nutrient transporters and chaperones, SLC1A5, SLC3A2, and SLC7A5 play a major role in driving the uptake of glutamine and leucine, which are critical for metabolic activation and cellular function (9–11). Thus, understanding the molecular regulation of these SLC transporters will provide insight into novel strategies to potentiate the metabolic capabilities and effector functions of tumor-directed engineered-NK and T cells in the TME.

## The Tumor Microenvironment: A Battlefield With Limited Nutrients

### Nutrient Depletion in the TME

Despite their heterogeneity, most cancers are uniformly characterized by an increase in metabolism and mitochondrial respiration (1). Metabolic changes seen in tumors are driven by either oncogenic mutations in cell signaling genes, changes in metabolic enzymes, or environmental factors (1, 12, 13). Since cancer cells constantly proliferate, they undergo Warburg metabolism or aerobic glycolysis, where they reduce the majority of the pyruvate into lactate instead of metabolizing to carbon dioxide in the mitochondrial tricarboxylic acid (TCA) cycle. By doing so, excess carbons may be used for the synthesis of lipids, proteins, and nucleotides and the production of new cells (1, 14). Furthermore, cancer cells strengthen their resistance to TILs partly through the metabolites released into the TME, contributing to its hostility. For example, numerous cancer cells and tumor-associated cells in the TME secrete or express factors like prostaglandin E2 (PGE2) and indoleamine 2,3-dioxygenase (IDO), which can inhibit the activation and metabolism of NK and T cells (1, 10). For instance, the increased expression of IDO and tryptophan-2,3-dioxygenase (TDO) by tumor cells, tumor-associated macrophages (TAMs) and tumor-associated dendritic cells and fibroblasts, which catalyze the conversion of tryptophan to kynurenine, results in tryptophan depletion and contributes to the dysfunction of TILs. The produced kynurenine is secreted by these cells, enters the TILs through the System L transporter, SLC7A5, and inhibits NK cell and T cell proliferation and effector function. It is a double-edged sword, as it suppresses NK cell and T cell activity as well as enhances the function of Tregs and myeloid-derived suppressor cells (MDSCs) (10, 14, 15).

Solid tumors lack a proper supply of nutrients and oxygen from blood vessels. As the tumor mass grows, newly formed blood vessels are generated to provide the cancer cells with oxygen and essential nutrients. However, tumor vascularization is disorganized in comparison to normal vasculature and, as a consequence, cells in the TME that are more distant from a blood vessel will be subjected to limited nutrients and reduced oxygen supply (1, 10, 14). The activation of aberrant metabolic pathways and stress response genes in cancer cells allows them to increase their uptake of major carbon sources, especially glucose and glutamine, and essential amino acids while maintaining energy production under hypoxic conditions to support their proliferation and anabolic requirements. Hypoxia-inducible factor (HIF)-1-mediated metabolic transformation is critical for the adaptation to hypoxia by increasing the expression of nutrient transporters, even though its activation is also induced by TCR and cytokine stimulation (16, 17). Several reports indicate that HIF-1 activation in T cells leads to enhanced control of persistent LCMV infection and neoplastic growth (18–20). However, in mice studies with conditional HIF-1α deficiency, HIF-1α suppresses the NF-κB signaling pathway in NK and T cells, affecting their effector functions (21, 22). Moreover, HIF-1α knock-down in CD8+ T cells improved the polyfunctionality of tumour-infiltrating CD8+ T cells and delayed tumor progression when these cells were adoptively transferred into tumor-bearing mice (23). Therefore, these data suggest that the role of HIF-1-mediated metabolic adaptation in the anti-tumor functions of NK and T cells is not fully elucidated and remains an important issue that needs to be addressed.

Since the metabolic processes in immune cells are linked to their effector functions, the harsh conditions in the TME limit the metabolic fitness of TILs, which can negatively impact their immune response against the cancer cells (1, 10, 24). For example, in ovarian cancer, glucose restriction was shown to impair CD8+ T cell survival and function due to the high expression of miR-101 and miR-26a, which repressed the methyltransferase EZH2 (25). EZH2 is important for the anti-tumor function of T cells by inducing cytokine expression and enhancing survival. Glucose addition was able to rescue the expression of EZH2 and reverse this phenotype (25). Interestingly, CD4+ T cells exposed to ovarian cancer ascites had severely reduced levels of the glucose transporter GLUT1, leading to reduced glucose uptake and defective N-linked protein glycosylation (26). The resulting defect in N-linked protein glycosylation caused endoplasmic reticulum (ER) stress by activating the unfolded protein response (UPR) *via* the IRE1α–XBP1 pathway. Upon ER stress, IRE1α induces the splicing of XBP1 mRNA, and the resulting isoform, XBP1s, activates genes that participate in protein folding. The IRE1α–XBP1 pathway was upregulated in T cells within ovarian cancer ascites, demonstrating that T cells undergo ER stress in the TME. Interestingly, a study in mice demonstrated that XBP1 induction in CD4+ T cells inhibited the expression of the glutamine transporters SLC1A5, SNAT1, and SNAT2 under glucose deprivation, leading to reduced glutamine uptake and oxidative phosphorylation, which limited IFNγ production. These results suggest that stress-inducing conditions in the TME can force the immune cells to reduce their expression of nutrient transporters and inhibit their nutrient uptake, therefore paralyzing them from accomplishing their effector functions (1, 26).

Lack of glutamine and glucose in the TME may shift ratios of T cell subsets by supporting the development of regulatory T cells (Treg) rather than effector T cells such as T helper 1 (Th1) cells and Th17 cells (1, 27, 28). For example, the overexpression of SLC1A5, SLC3A2, and SLC7A5 in breast cancer is significantly associated with the existence of Foxp3+ Tregs and poor patient survival (29). Treg cells and other infiltrating regulatory cells, such as highly anabolic tolerogenic dendritic cells, also compete for the nutrients and contribute to the nutrient-limited TME. In addition, Treg cells produce adenosine from ATP, which suppresses immune cell activity *via* A_2A_, an adenosine receptor that suppresses IL-2 production (1, 30).

### SLC1A5, SLC3A2 and SLC7A5

Numerous studies have investigated the amino acid exchangers comprised of SLC1A5 and SLC7A5 with the ancillary subunit SLC3A2; these three proteins are among the highest differentially expressed genes in activated lymphocytes and cancerous cells (1, 13, 31–35). SLC3A2, also known as CD98 or 4F2 heavy chain (4F2hc), is a type II membrane protein. SLC3A2 dimerizes with several light chains of nutrient transporters, such as SLC7A5, also known as LAT1, to act as a chaperone and allow their localization to the plasma membrane (33, 36). A report demonstrated that the SLC3A2/SLC7A5 heterodimer is an amino acid exchanger that functions in conjunction with SLC1A5, a sodium dependent antiporter also known as ASC amino acid Transporter 2 (ASCT2) (35). In this model, glutamine serves as a major substrate of the SLC3A2/SLC7A5 bidirectional transport for the uptake of essential amino acids (EAAs) such as L-leucine and L-tryptophan. However, glutamine is a substrate with a very low affinity for SLC3A2/SLC7A5 reconstituted on proteoliposomes (37), therefore the critical role of glutamine in driving the transport of L-leucine and EAAs *in vivo* remains to be determined. Notably, in addition to the role of amino acids as cellular building blocks or fuels, some of these EAAs like L-leucine and L-arginine can function as signaling molecules for mTORC1 activation (33). It has been shown that the abrogation of these nutrient transporters negatively impacts the effector functions of NK and T cells. For instance, the deletion of SLC3A2 prevented T cell expansion, while the deletion of SLC7A5 prevented T cell effector differentiation, mTORC1 activation, and c-Myc expression (38–40). Deletion of SLC7A5 also prevented the expansion of CD4 T cells and the release of certain proinflammatory cytokines in mouse models of skin inflammation (40). In addition, SLC7A5 and SLC1A5 deficient mice have defective metabolism and activation of mTORC1 (34, 41). Moreover, pharmacological inhibition of SLC1A5 and SLC3A2 was found to abrogate the effector functions of NK cells, and inhibition of SLC7A5 in cytokine-activated NK cells resulted in reduced c-Myc protein levels and mTORC1 signalling (24, 31).

## Molecular Regulation of SLC1A5, SLC3A2, and SLC7A5

### mTOR

**Figure 1:**
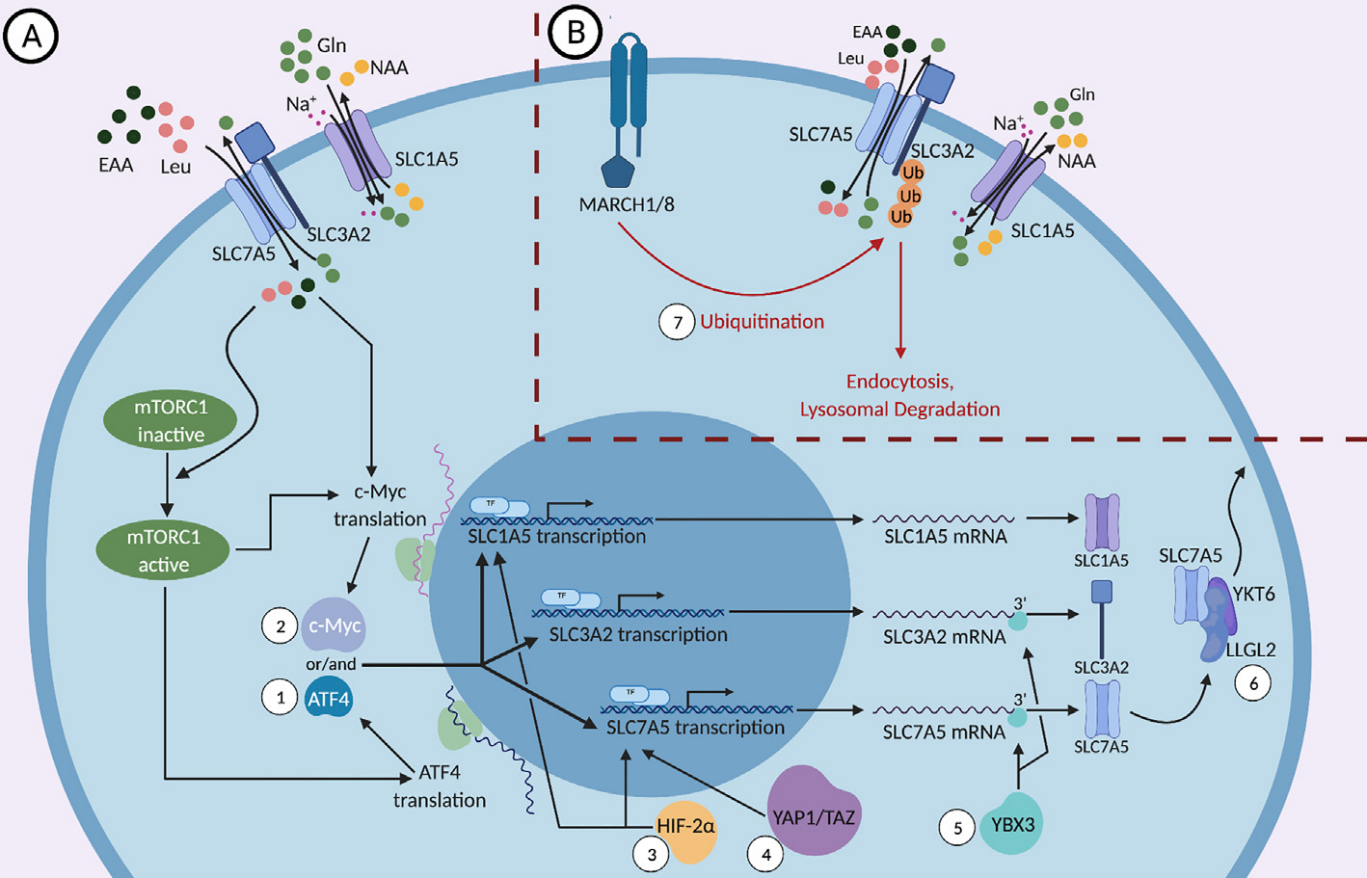
**(A)** Positive and **(B)** negative molecular regulations of SLC1A5, SLC3A2, and SLC7A5. SLC3A2, a type II membrane protein, dimerizes with the nutrient transporter SLC7A5 to allow their localization to the plasma membrane. SLC1A5, the sodium dependent antiporter exchanges neutral amino acids (NAA) such as threonine, asparagine or serine for glutamine and the SLC3A2/SLC7A5 heterodimer exchanges glutamine for essential amino acids (EAAs), most importantly L-leucine. While the mTOR signaling pathway regulates the expression of SLC1A5, SLC3A2, and SLC7A5, these proteins can activate mTOR in a feed-forward mechanism through the influx of essential amino acids (EAAs), especially leucine. (1) mTORC1 regulates the translation and stability of the mRNA encoding the transcription factor ATF4, which in turn controls the transcription of *SLC1A5, SLC3A2*, and *SLC7A5*. (2) mTORC1 and the influx of EAAs regulate the expression of the transcription factor c-Myc, which binds the promoters of *SLC1A5* and *SLC7A5* genes and upregulates their expression. (3) HIF-2α, a transcription factor that is activated in response to hypoxic conditions, binds to the promoter of *SLC1A5* and *SLC7A5* and activates their transcription. (4) YAP1 and TAZ bind to the promoter of *SLC7A5* and activate its transcription. (5) The DNA- and RNA-binding protein YBX3 enhances the stability of *SLC7A5* and *SLC3A2* transcripts by binding directly to their 3’ UTRs and prevents their degradation. (6) Under nutrient stress, LLGL2 forms a trimeric complex with SLC7A5 and a regulator of membrane fusion, YKT6, allowing for the surface localization of SLC7A5 and thereby promoting cellular proliferation. (7) MARCH1 and MARCH8 ubiquitin ligases lead to the direct ubiquitination of SLC3A2 and its degradation by endosomes and lysosomes.

The signaling pathways that regulate the metabolism of immune cells, specifically NK and T cells, are a major focus of research because they are linked to and are essential for their effector functions. mTOR, a conserved serine/threonine kinase, is a central metabolic regulator that promotes cellular growth, proliferation, and survival. There are two main mTOR complexes, mTORC1 and mTORC2, which participate in distinct cellular processes. mTORC1 is essential for the metabolic reprogramming that is critical for NK and T cell effector functions (42–44). mTORC1 increases the expression of SLC1A5, SLC3A2, and SLC7A5 by regulating the translation and stability of the mRNA encoding the transcription factor ATF4 (45, 46). ATF4 controls the expression of several amino acid transporters, including SLC1A5, SLC3A2, and SLC7A5, as well as other metabolic enzymes. It was shown that mTOR inhibition reduces ATF4 levels as well as the levels of mRNAs targeted by ATF4 (**Figure 1**), leading to a reduction in SLC3A2 and SLC7A5 expression (45).

The mTOR signaling pathway is a two-way street because mTOR activity is affected by SLC1A5, SLC3A2, and SLC7A5 expression in a feed-forward fashion in addition to regulating their expression (**Figure 1**). Since certain EAAs are important for the induction of mTOR (35, 47), the expression of nutrient transporters is critical to ensure proper nutrient uptake required for optimal mTORC1 activation. It has been shown that the upregulation of both the System L amino acid transporter SLC7A5, and the glutamine-transporter SLC1A5, is essential for mTORC1 activity (34, 35, 48, 49). These transporters allow for the influx of amino acids such as leucine, which in turn activates the nutrient-sensing Rag GTPases, enzymes upstream of mTORC1 (48, 50–52). It is important to note that leucine, arginine and other EAAs are sensed by both cytosolic and lysosomal sensors and play a key role in mTORC1 activation (50, 52–56). Under glucose restriction, mTORC1 activity is negatively regulated by adenosine monophosphate-activated protein kinase (AMPK) (47). Hence, in the TME, the reduced availability of nutrients predisposes cytotoxic immune cells to sub-optimal activation of mTORC1, which can negatively impact the metabolic fitness of the cells and reduce their anti-tumor activity.

### c-Myc Expression

c-Myc was shown to bind the promoters of SLC1A5 and SLC7A5 genes and upregulate their expression in cancer cells (**Figure 1**), indicating that c-Myc acts to sustain amino acid uptake through these transporters (57–59). c-Myc is essential for NK cell metabolism and effector function upon IL-2/IL-12 stimulation as well as for CD3/CD28 mediated metabolic reprogramming and activation of T cells (24, 60). NK cells lacking c-Myc have reduced metabolic activation upon cytokine stimulation, characterized by reduced glycolysis and mitochondrial respiration, accompanied by reduced IFNγ production and granzyme B expression (24). In T cells, c-Myc expression is required for activation-induced glycolysis and glutaminolysis, and glutamine uptake is crucial for T cell proliferation (59). Interestingly, amino acid transport through SLC7A5 and glutamine uptake through SLC1A5 were a requirement for c-Myc induction in cytokine-stimulated NK cells, however the presence of leucine was not required (24). The glutaminolysis pathway converts the imported glutamine into glutamate and then into α-ketoglutarate to feed into the TCA cycle and support oxidative phosphorylation. In NK cells, glutaminolysis was not required for the upregulation of c-Myc, even though glutamine uptake was necessary for c-Myc upregulation (24). This suggests that glutamine induces c-Myc expression through its exchange with essential amino acids other than leucine *via* the system L transporter. In summary, amino acid transport upregulates c-Myc, which then stimulates the expression of the nutrient transporters SLC1A5 and SLC7A5, as well as the ancillary subunit SLC3A2 (**Figure 1**), in a positive feedback fashion, enhancing the uptake of EAAs to sustain mTORC1 activity and further support c-Myc expression (59). Notably, mTORC1 was required for the early induction of c-Myc in NK cells, but was not necessary to maintain a sustained expression of c-Myc (24).

### Regulation in Hypoxic Conditions

Another transcription factor that binds to the promoter of SLC7A5 is HIF-2α, a factor that regulates transcriptional responses during hypoxic conditions in most mammalian cells (**Figure 1**). Through the upregulation of SLC7A5, HIF-2α is able to increase mTORC1 activity (61). This confers an advantage to tumor cells in the hypoxic, nutrient-deficient TME and supports their proliferation and growth. HIF-2α has also been shown to induce the expression of a mitochondrial variant of SLC1A5 under hypoxic conditions (62). This variant of SLC1A5, transcribed from a different transcription initiation site and carrying an N-terminal signal for localization to the mitochondria, was recently discovered in pancreatic cancer cells (62). Overexpression of this variant enhanced cellular metabolism and ATP production and increased gemcitabine resistance, which would otherwise have a significant impact on cancer cell growth in normoxia (62, 63). Consistently, knockdown of the mitochondrial variant of SLC1A5 variant in cancer cells leads to drastic tumor inhibition *in vivo*. It is important to note that HIF-2α is also involved in the regulation of other amino acid transporters, such as SLC1A1, SLC1A3 and SLC38A2 as shown in certain tumor cells, that may impact cell activity in hypoxic environments (64–66).

### Cytokine-Mediated Upregulation

Pro-inflammatory cytokines that activate the effector functions of lymphocytes tend to induce the upregulation of proteins involved in amino acid transport, including SLC1A5, SLC3A2, and SLC7A5. For instance, IL-2 stimulation of CD8 T cells was shown to increase SLC7A5 expression, which was sustained with continuous cytokine exposure (39, 67). Moreover, IL-2 upregulates SLC1A5 and SLC3A2/SLC7A5 in NK cells, and these transporters are needed for IFNγ production and degranulation (31). IL-15 is one of the most potent cytokines in enabling NK cell homeostasis and activation through the mTOR pathway. During microbial infections, NK cells stimulated with IL-15 display enhanced responses as well as increased IFNγ production. SLC1A5, SLC7A5, and SLC3A2 expression were increased upon treatment with IL-15 and, to a lesser extent, upon IL-12 treatment. IL-2 and IL-15 induce SLC1A5 and SLC3A2/SLC7A5 expression *via* the JAK3/STAT5 pathway (31, 68–70). Stimulation with IL-7 also resulted in increased SLC1A5 and SLC7A5 expression in CD8+ T cells (41). Recently, we have shown that IL-18 is a key cytokine that can induce a robust upregulation of these three amino acid transporters in NK cells, consequently inducing leucine-driven mTORC1 activation and metabolic transformation, leading to enhanced proliferation and effector function (9).

### Ubiquitination

One mechanism by which cells regulate their expression of nutrient transporters is the process of ubiquitination-mediated protein degradation. SLC3A2 trafficking is ubiquitin-dependent, and multiple residues within SLC3A2 and SLC7A5 were identified as ubiquitination sites (33). Ubiquitination is the addition of the small protein ubiquitin or a chain of ubiquitin to substrates *via* enzymatic processes. MARCH1 and MARCH8 are transmembrane proteins that are mostly limited to immune cells and that catalyze the ubiquitination of certain substrates; however, MARCH8 was also shown to be expressed in esophageal squamous cell carcinoma and is associated with tumor growth (71–73). The surface expression of SLC3A2 is downregulated when MARCH1 and MARCH8 are overexpressed (**Figure 1**). Those ubiquitin ligases lead to the direct ubiquitination of SLC3A2 and its degradation by endosomes and lysosomes (71). It was shown that when a ubiquitination-resistant-mutant form of SLC3A2 was expressed in T cells, cell proliferation and clonal expansion increased (74), although this might be due to the role of SLC3A2 not directly linked to its transport function (75). Moreover, knockdown of MARCH8 in HepG2 cells, a human liver cancer cell line, enhanced the expression of SLC3A2 and the iron transporter CD71 (76). A recent study showed that a deficiency in MARCH1 causes heightened NK cell activation and production of proinflammatory cytokines upon stimulation with LPS (72, 77). The expression of MARCH proteins can be regulated by extracellular stimulation. For example, TNFα stimulates the upregulation of MARCH1 and results in further downregulation of the SLC nutrient transporters (72). Thus, the expression of MARCH1 and MARCH8 may contribute to maintaining the homeostasis of immune cells during inflammation.

Other enzymes are also involved in the ubiquitination of the nutrient transporters and their downregulation. For example, the Nedd4-2 ubiquitin ligase was found to ubiquitinate the N-terminal tail of SLC7A5 and elicit its downregulation and endocytosis upon activation of protein kinase C by phorbol 12-myristate 13-acetate (PMA) (78). Another example is the ubiquitin-editing enzyme A20, which has been shown to regulate mTOR activity in NK and T cells. Upon deletion of A20, mTOR activity increased in both NK and T cells and SLC3A2 expression was shown to be elevated in NK cells. However, whether only SLC3A2 is regulated by A20 or if transporters such as SLC1A5 and SLC7A5 are also targets of A20 is unknown (79).

### Regulation Observed in Non-Immune Cells

The Y-box (YBX) protein family, comprised of the three genes, YBX1, YBX2, and YBX3, is associated with cellular processes like cell proliferation and inflammatory diseases (80). In a recent study, the DNA- and RNA-binding protein YBX3 was required to maintain physiological levels of *SLC7A5* and *SLC3A2* mRNAs. Knockdown of YBX3 in HeLa cells decreased the levels of transcripts encoding *SLC7A5* and *SLC3A2*, leading to reduced protein expression. These reductions in amino acid transporter expression resulted in a reduced influx of EAAs at a steady state. YBX3 was shown to enhance the stability of *SLC7A5* and *SLC3A2* transcripts by binding directly to the 3’ UTR (**Figure 1**). A *SLC7A5* transcript that lacks the 3’ UTR was stable in the absence of YBX3, suggesting that the 3’ UTR contains a sequence that guides the mRNA for degradation and that YBX3 protects the sequence to prevent the transcript from degradation (80).

Another example of a protein that regulates the expression of nutrient transporters is LLGL2. The mammalian homologues of Drosophila Lgl, LLGL1, and LLGL2, are proteins that regulate scaffolding and epithelial cell polarity. In estrogen receptor (ER) positive breast cancers, LLGL2, but not LLGL1, is overexpressed, and its high expression correlates with poor patient survival (81). Notably, estrogen signaling induces LLGL2 expression, and LLGL2 was required for estrogen-mediated proliferation of breast cancer cells in low nutrients conditions (limited concentrations of glutamine and leucine). Under nutrient stress, as is the case in the TME, LLGL2 was shown to form a trimeric complex with SLC7A5 and a regulator of membrane fusion, YKT6. Indeed, LLGL2 was required for the surface localization of SLC7A5, suggesting that LLGL2 promotes tumor growth by upregulating nutrient transporters to enhance nutrient uptake within a nutrient-limited microenvironment (**Figure 1**). It is believed that ER+ patients develop resistance to tamoxifen treatment because of the LLGL2/SLC7A5-dependent adaptation to nutrient stress (81). Whether these regulatory pathways also operate in immune cells remains to be determined.

Finally, YAP1 and TAZ, downstream effector proteins of the Hippo tumor suppressor pathway, are also examples of proteins that upregulate SLC7A5 expression. The Hippo pathway is seen to be downregulated in many cancer cells and when suppressed, the downstream proteins YAP1 and TAZ promote transcription of genes, like *SLC7A5*, that enhance cell proliferation. It has been shown that YAP1 and TAZ bind directly to the *SLC7A5* promoter to upregulate its transcription and inhibition of SLC7A5 blocks YAP1/TAZ-mediated tumorigenesis of hepatocellular carcinoma. Through the upregulation of SLC7A5, YAP1/TAZ are able to increase mTORC1 activity and tumor proliferation and survival (82).

## Manipulating the Amino Acid Transporters for Cancer Immunotherapy

### Inhibiting the SLC Transporters for the Treatment of Cancers

Since SLC1A5, SLC3A2, and SLC7A5 are important for the metabolism, growth, and proliferation of cancer cells, these transporters have been targeted pharmacologically to block cancer cell growth and survival (46, 83–88). One inhibitor that has shown a favorable safety profile and modest evidence of anti-tumor activity in phase I clinical trial for acute myeloid leukemia (NCT02040506) is IGN523, a humanized anti-CD98 (anti-SLC3A2) monoclonal antibody (89). Preclinical studies have demonstrated that IGN523 exhibits potent anti-tumor activity *in vivo* in several xenograft models such as patient-derived lymphoma and non-small-cell lung carcinoma (90, 91). Moreover, the LAT-1 (SLC7A5) inhibitor JPH203 showed encouraging results in phase I clinical trial to treat advance solid tumors (UMIN000016546) and is currently used in a placebo-controlled randomized phase II study (UMIN000034080). The study showed that treatment with JPH203 could achieve partial response in one patient with biliary tract cancer who continued treatment for two years without showing signs of disease progression (91). In addition, SLC7A5 deletion in human colon, lung, and kidney cancer cell lines resulted in mTORC1 inhibition leading to tumor growth arrest *in vitro and in vivo*. Interestingly, SLC3A2 deletion in the same cell types showed intact mTORC1 activity and tumor growth rate, but the cells were sensitive to SLC7A5 inhibition *via* treatment with JPH203. Double knockout of SLC3A2 and SLC7A5 in these cells resulted in a greater reduction of mTORC1 activity and *in vitro* proliferation. This indicates that residual SLC7A5 activity may allow normal cell function in SLC3A2-deficient tumor cells. Also, this shows that treatment of cancers with a SLC7A5 inhibitor or a combination of SLC7A5 and SLC3A2 inhibitors would be more effective than treatment with SLC3A2 inhibitor alone (92).

Although there is currently no SLC1A5 inhibitor being tested in clinical trials, a few SLC1A5 inhibitors have shown promising results in preclinical studies (93). One example is V-9302, which was shown to increase cell death and abrogate cancer cell growth *in vitro* and *in vivo* (94). Interestingly, other studies have shown that SLC1A5 suppression or deletion fails to prevent tumor growth, but this discrepancy could be due to the effect of the mitochondrial variant of SLC1A5, which was previously overlooked (62, 63, 95). Altogether, developing inhibitors that target nutrient transporters, including the mitochondrial SLC1A5 variant, is a promising new approach to weaken the cancer cell metabolism and reduce tumor growth. However, such inhibitors would likely target the nutrient transporters on immune cells and thereby weaken the immune response simultaneously; therefore, developing inhibitors that target the nutrient transporters specifically on cancer cells would be ideal. One exceptional example is that pharmacological inhibition of glutamine uptake using the glutamine transporter inhibitor, V-9302, selectively blocked glutamine uptake by triple-negative breast cancer cells but not CD8+ T cells. Interestingly, CD8+ T cells use a compensatory pathway to upregulate an alternative glutamine transporter, SLC6A14, and sustain glutamine uptake and effector function in V-9302-treated tumors (96).

### Targeting Immune Cells for the Upregulation of SLCs

**Figure 2:**
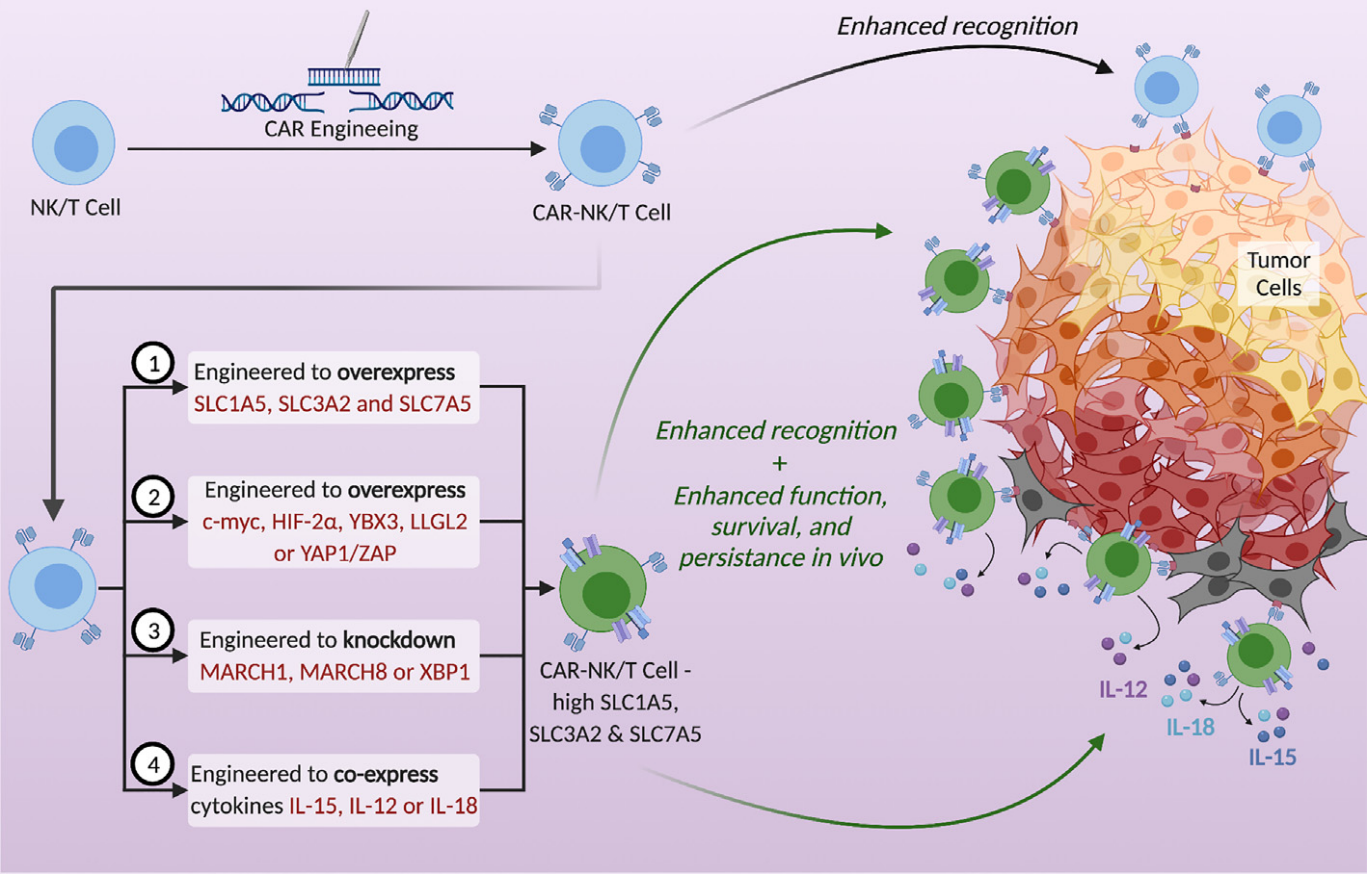
Potential strategies to utilize SLC1A5, SLC3A2, and SLC7A5 to enhance the metabolic fitness of NK and T cells and thereby strengthen anti-tumor immunotherapy. CAR-NK/T cells exhibit enhanced tumor recognition but are often metabolically disadvantaged in the TME. Upregulation of the SLC transporters in CAR-NK/T cells using the proposed tactics could enhance their function, survival, and persistence *in vivo*. Possible strategies are (1) to genetically engineer CAR- NK/T cells to overexpress these transporters, (2) to overexpress specific proteins that increase the expression of SLCs, (3) to knockdown genes that decrease the expression of SLCs, and (4) to engineer CAR-NK/T cells to co-express cytokines, specifically IL-15, IL-12, or IL-18.

The adoptive transfer of genetically engineered T cells or NK cells expressing chimeric antigen receptors (CARs) that increase the specificity of immune cells against the tumor is an attractive treatment plan in cancer therapy. However, these cells are still at a disadvantage because they need to compete with cancer cells for nutrients within the TME. Moreover, these CAR-modified immune cells lack efficacy against solid tumors. A large area of research is currently focused on enhancing the function and maximizing the efficacy of CAR-NK and T cells by navigating the metabolic barriers involved (14). For example, one study looked into engineering the CAR-T cells to also express 4-1BBL, a ligand that enhances T cell persistence by stimulating dendritic cells to release supportive cytokines (14, 70, 97, 98). Another example is engineering CAR-NK cells to secrete IL-15 to support NK cell function in an autocrine fashion (99–101). A novel way to enhance CAR-NK and T cell function is to upregulate the SLC1A5, SLC3A2, and SLC7A5 transporters (**Figure 2**). Since their upregulation is needed for immune cell proliferation and effector function, engineering NK or T cells to overexpress these amino acid transporters could be a promising direction in immunotherapy. Such manipulations could potentially strengthen the metabolic fitness of NK and T cells and become a new approach to cope with the nutrient stress suffered in the TME, thereby improving anti-tumor immunotherapies. According to the mechanisms of regulation of the nutrient transporters expression as discussed above, potential strategies to enhance the expression of SLC proteins in immune cells also include the overexpression of positive regulators and the deletion or knockdown of negative regulators (**Figure 2**). Proteins to overexpress include c-Myc and HIF-2α to activate the promoters of *SLC1A5* and *SLC7A5*, YAP1/TAZ to activate the promoter of *SLC7A5*, the RNA-binding protein YBX3 to stabilize *SLC3A2* and *SLC7A5* transcripts, and LLGL2 to increase surface localization of SLC7A5. Genes to delete or knockdown include those encoding the ubiquitination proteins like MARCH1 and MARCH8 to upregulate SLC3A2, or XBP1 to upregulate the glutamine transporters SLC1A5, SNAT1 and SNAT2. Moreover, cytokine stimulation can be used to upregulate SLC transporters expression. Many important cytokine receptors, including those for IL-15, IL-12 or IL-18 are predominantly expressed on immune cells and are absent on non-hematopoietic cells. Therefore, cytokines can be harnessed to specifically enhance SLC transporters expression and immune cell effector functions, while having little effect on cancer cells of non-hematopoietic origin. Arming the CAR-NK and T cells by engineering them to upregulate SLC1A5, SLC3A2 and SLC7A5, or to secrete cytokines in a controlled manner, could be a potential solution to overcoming the nutrient deficiency in the TME. By doing so, NK and T cells will have a metabolic advantage through their increased ability to uptake glutamine, leucine and other amino acids essential for their growth, proliferation and effector functions.

## Of Interest – Possible Roles in COVID-19

Severe acute respiratory syndrome coronavirus 2 (SARS-CoV-2) is the novel virus that is causing the mayhem of the COVID-19 pandemic worldwide. The SARS-CoV-2 invades cells through its Spike Protein (SP), which binds to ACE2 or the recently discovered CD147 on the host cell (102, 103). CD147 is expressed on the surface of immune cells, including NK cells and CD4+ and CD8+ T cells, and the SARS-CoV-2 can invade these cells through the SP-CD147 route (102, 104–106). In a recent clinical trial, meplazumab, a humanized antibody targeting CD147, was used to treat coronavirus patients, resulting in improved clinical outcomes (107). Interestingly, CD147 forms a super-complex with CD98hc (SLC3A2) and thus is associated with the system L amino acid transporter. Moreover, knockdown of CD98hc caused a depletion of CD147 (108). Therefore, perhaps manipulating CD98 levels may provide other methods to treat the new SARS-CoV-2.

## Conclusion

In conclusion, the amino acid exchangers, SLC1A5 and SCL3A2/SLC7A5 heterodimeric complex, are necessary for efficient uptake of essential amino acids and immune cell metabolism. They can also activate mTORC1, a metabolic regulator that promotes cell metabolism and c-Myc, which promotes cell growth, proliferation, and survival. Cancer cells have a metabolic advantage and are superior in the uptake of nutrients. They also create a hostile TME that makes it difficult for the cytotoxic immune cells to adapt, infiltrate the tumors, survive and defeat cancerous cells. Current anti-tumour immunotherapies like CAR-NK/T cells, which enhance tumor recognition, are often metabolically disadvantaged in the nutrient deficient and hostile TME. Manipulating such therapies to increase SLC1A5, SLC3A2, and SLC7A5 expression in the immune cells could enhance anti-tumor immunotherapy and lead to developments in the field. Engineered NK and T cells could be modified to overexpress these nutrient transporters or to co-express immune-stimulatory cytokines. Upregulation of the SLC transporters in engineered NK and T cells using the proposed tactics could increase their effectiveness, function and survival *in vivo*, leading to better prognoses in patients.

## Author Contributions

MN, AA, SA, and S-HL wrote the manuscript. MN prepared the figures. All authors contributed to the article and approved the submitted version.

## Funding

This work was supported by funding from the Canadian Institutes of Health Research (PJT-156106) to S-HL. The University of Ottawa covered 50% of the article processing charge.

## Conflict of Interest

The authors declare that the research was conducted in the absence of any commercial or financial relationships that could be construed as a potential conflict of interest.
